# Trends in cognitive function before and after myocardial infarction: findings from the China Health and Retirement Longitudinal Study

**DOI:** 10.3389/fnagi.2024.1283997

**Published:** 2024-02-22

**Authors:** Jing Shang, Jianye Dong, Sijia Zhu, Qingmei Chen, Jianian Hua

**Affiliations:** ^1^Department of Psychiatry, The First Affiliated Hospital of Soochow University, Suzhou, Jiangsu, China; ^2^Department of Obstetrics and Gynecology, The First Affiliated Hospital of Soochow University, Suzhou, Jiangsu, China; ^3^Department of Neurology, The Fourth Affiliated Hospital of Soochow University, Medical Center of Soochow University, Suzhou Dushu Lake Hospital, Suzhou, Jiangsu, China; ^4^Department of Physical Medicine and Rehabilitation, The First Affiliated Hospital of Soochow University, Suzhou, Jiangsu, China; ^5^Department of Neurology, The First Affiliated Hospital of Soochow University, Suzhou, Jiangsu, China

**Keywords:** myocardial infarction, coronary artery disease, cognitive function, cohort study, aging

## Abstract

**Objectives:**

Incident stroke was associated with cognitive dysfunction after stroke and even before stroke. However, cognitive trends prior to myocardial infarction (MI) and the timeline of cognitive decline in a few years following incident MI remain unclear, especially among the Chinese population. We aimed to evaluate whether MI was associated with cognitive change both before and after MI in China.

**Methods:**

This cohort study included 11,287 participants without baseline heart problems or stroke from the China Health and Retirement Longitudinal Study. The exposure was self-reported MI. The outcomes were scores of cognitive functions in five domains, which reflected abilities of episodic memory, visuospatial abilities, orientation, attention and calculation, and global cognition as a summary measure. A Linear mixed model was constructed to explore cognitive function before and after incident MI among the MI participants and the cognitive trends of participants free of MI.

**Results:**

During the 7-year follow-up, 421 individuals [3.7% of 11,287, mean (SD) age, 60.0 (9.0) years; 59.1% female] experienced MI events. The cognitive scores of participants of both the MI group and the control group without MI declined gradually as time went by. The annual decline rate of the MI group before incident MI was similar to that of the control group during the whole follow-up period. Incident MI was not associated with acute cognitive decline in all five cognitive domains. Moreover, MI did not accelerate the cognitive decline rate after MI compared with the pre-MI cognitive trends. The decline rate of cognitive function after MI was similar to the rate before MI.

**Conclusions:**

Different from stroke, participants who had an MI did not show steeper cognitive decline before MI. MI was not associated with acute cognitive decline and accelerated decline in several years after MI. Future studies are needed to learn the mechanisms behind the different patterns of cognitive decline between MI and stroke.

## 1 Introduction

Dementia is becoming a dire global health concern (Wimo et al., 2017). Mild cognitive impairment (MCI) is a stage of cognitive impairment beyond normal aging but does not meet the diagnostic criteria for dementia (Winblad et al., 2004). MCI is associated with a 10% to 16% annual risk of developing dementia and is recognized as a therapeutic window for dementia (Farias et al., 2009; Tsoi et al., 2017). Considering that both dementia and MCI are characterized by declines in cognitive function, a comprehensive understanding of the risk factors for cognitive decline might help reduce the huge burden of dementia on families and societies.

Myocardial infarction (MI) has previously been linked to cognitive impairment (Gorelick et al., 2011; Kasprzak et al., 2021). Survivors of MI is growing thanks to advances in treatment and population aging (Heidenreich et al., 2011; Levine et al., 2014). The most common forms of dementia are Alzheimer's disease (60%−80%), vascular dementia (7%−25%), frontotemporal dementia, and Lewy body dementia (World Health Organization, 2012). MI affects brain health mainly through vascular-dementia-related pathology (Sundboll et al., 2018). Heart failure and low perfusion after MI directly impair cognitive function (Wolters et al., 2018; Vishwanath et al., 2022). MI was indirectly associated with cognitive impairment through clinical or subclinical stroke, which has a high incidence among MI survivors (de la Torre, 2022; Koton et al., 2022). Moreover, cardiovascular risk factors, such as hypertension, diabetes, and even the synergistic effect of both, were correlated with MI and cognitive impairment. However, whether the cognitive impairment is due to the cardiovascular event itself or to the cardiovascular risk factors (e.g., hypertension, diabetes) remains unelucidated (Gottesman and Johansen, 2021). The cognitive decline caused by hypertension or diabetes is slow and progressive. If cardiovascular risk factors are the main indicator of cognitive decline, patients' cognitive function may have already declined even before MI onset (Zheng et al., 2019). Nevertheless, most studies assessed cognitive function after the onset of heart disease. For example, clinical doctors could only achieve the cognitive data during hospitalization or after discharge. Surgeries are interested in the risk of dementia after bypass graft surgery, but hard to ascertain the cognitive status before the surgery (Greaves et al., 2020). Cognitive function before MI onset is impossible to obtain.

The current study aimed to explore cognitive trends both before and after incident MI using a large nationally representative sample from the China Health and Retirement Longitudinal Study (CHARLS).

## 2 Materials and methods

### 2.1 Study population

Harmonized with the Health and Retirement Study (HRS), CHARLS is an ongoing nationally representative longitudinal survey in China. Its study population was middle-aged and elderly (aged ≥45 years) Chinese participants and their spouses, who were randomly selected from 150 counties within 28 provinces through multistage probability sampling. The CHARLS collects economic, social, and health data to deal with the challenges posed by China's population aging. The first survey (Wave 1) of CHARLS was conducted between 2011 and 2012. Follow-up surveys were conducted at 2- to 3-year intervals till 2018. Ethics approval is shown in Supplementary Text 1. The comprehensive study design has already been covered elsewhere (Zhao et al., 2014).

**Figure 1:**
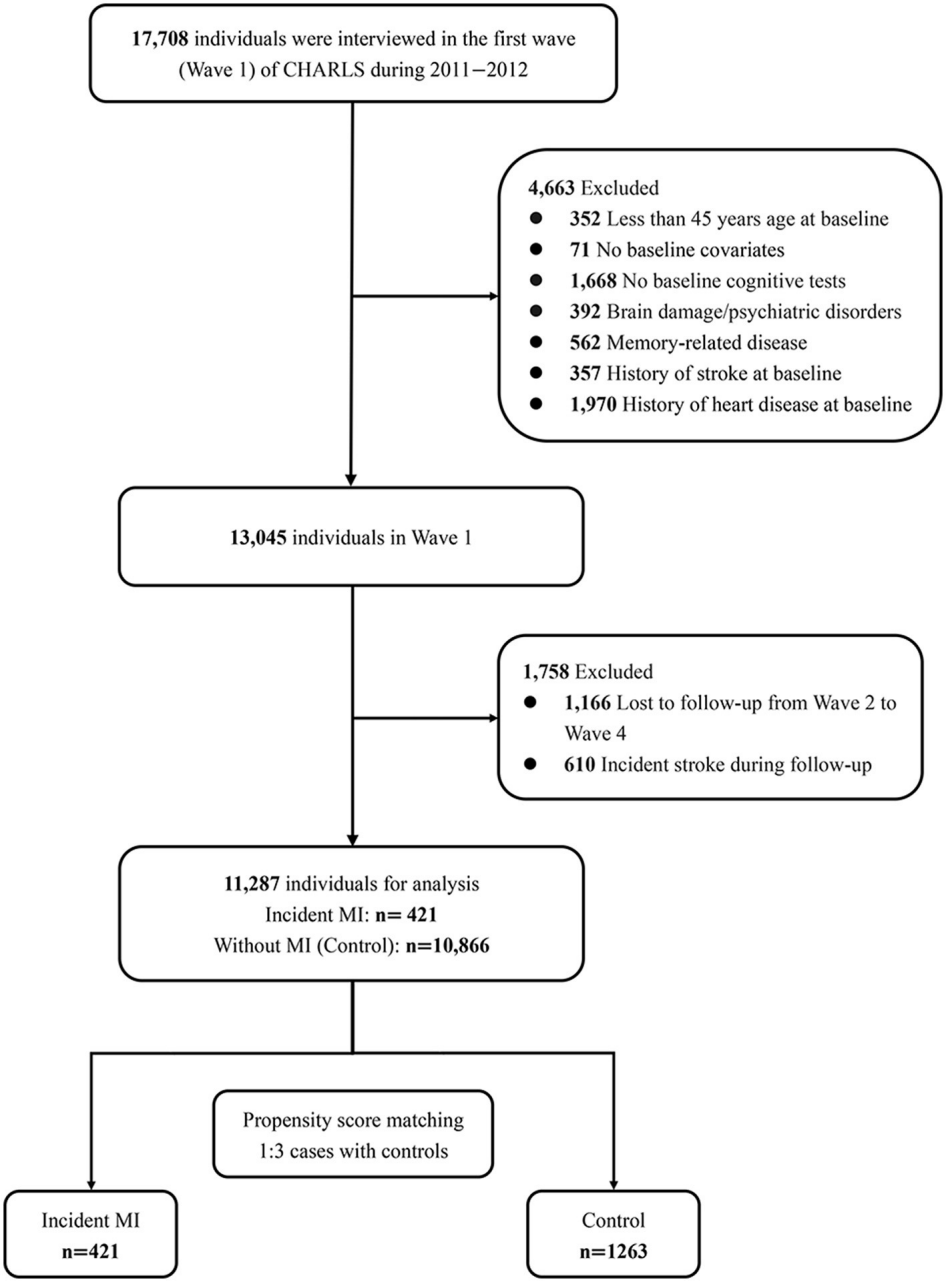
Flow chart of study participants.

Figure 1 presents the sample selection process. We defined Wave 1 of CHARLS as the baseline of the current research. Four thousand six-hundred sixty-three participants were excluded at baseline because of being under 45 years old (*n* = 352), missing baseline covariates (*n* = 71) or baseline cognitive tests (*n* = 1,668), having a history of memory-related disease (*n* = 562), brain damage (*n* = 392), stroke (*n* = 562), or heart disease (*n* = 1,970). Patients would experience pronounced cognitive decline in the short term after stroke (Zheng et al., 2019). We excluded participants with stroke to avoid confounding bias. Then, 1,758 of the remaining individuals were disqualified due to loss to follow-up (not completing at least one cognitive assessment from waves 2 to 4, *n* = 1,166) or experienced a stroke during follow-up (*n* = 610). Finally, 11,287 individuals were enrolled in the current study, including 421 participants who had an MI during the follow-up and 10,866 participants without MI.

### 2.2 Assessment of cognitive function

CHARLS collected participants' cognitive function at each visit (Wave 1–Wave 4). Four cognitive domains were assessed by different tests and the detailed methods are as follows: (1) Episodic memory: word recall test was utilized to estimate both immediate and delayed memory. The investigator read 10 words while participants should recall these words immediately and after 5 min. The total scores were 10. (2) Visuospatial ability: individuals were shown two overlapping pentagons and people who successfully replicated the drawing could get one point in the figure drawing test. (3) Orientation ability: It is assessed by answering the date (day, month, year), week, and season. The score ranged from 0 to 5. (4) Calculation and attention ability: participants were asked to perform five consecutive subtractions of 7 from 100 and each correct answer got 1 point (total score: 5). The effectiveness and dependability of these cognitive tests have been clearly demonstrated in the past investigations (Zhao et al., 2014; Meng et al., 2019; Hua et al., 2023).

In order to acquire a more intuitive comparison across different cognitive tasks, *z* scores were generated by the mean and SD of baseline scores. The *z*-score of global cognition, a summary of performance on the above four cognitive domains, was calculated by adding the *z*-scores of the four cognitive domains and then normalizing them to baseline in the same manner. Hence, a *z*-score of −1 indicates a cognitive score being 1 SD below the average baseline score.

### 2.3 Assessment of incident MI

In Wave 1, participants were asked whether they had been told by a doctor that they had heart problems, including MI, angina, CHD, congestive heart failure, and other heart problems (Zhao et al., 2014). No information about the type of heart problem is available. Those with heart problems at Wave 1 were excluded according to our selection criteria. From Wave 2 to Wave 4, MI was based on self or proxy reports of physician diagnosis. However, other types of heart disease were not recorded. Some participants reported the year of MI. The time of MI of other participants was defined as the midpoint of the last wave without MI and the first wave reporting MI.

### 2.4 Covariates

Details of the covariates are described in Supplementary Text 2 (Cheng and Chan, 2005; Zhao et al., 2014). Potential confounding covariates were chosen according to relevant literature (Zheng et al., 2019), and were associated with cognitive function or MI, which included age, sex, educational level, marital status, area of residence, smoking, drinking, number of instrumental activities of daily living (IADLs), depression, hypertension, diabetes, high total cholesterol, lung diseases, and cancer.

### 2.5 Statistical analysis

Baseline characteristics between participants with and without incident MI, and between participants included and excluded due to loss of follow-up were compared. Our primary analysis was a two-level linear model (Supplementary Figure 1). The model accommodated the repeated measurements of cognitive function over time and calculated the effect of MI and other covariates on the trajectories of cognitive function (Laird and Ware, 1982; West et al., 2007; Bell et al., 2013; Berglund, 2018). Fixed effects were fitted for intercept, baseline covariates, MI group (the group with incident MI and the control group without MI), time (years since baseline), “group^*^time” interaction, a time-varying MI status (whether or not had a history of MI at the current wave, 1 for with MI and 0 for without-MI), and “group^*^MI-status^*^years-after-MI” interaction. Random effects were fitted for intercept and time (years since baseline and years after MI). In subgroup analysis, we restricted the analysis to those with incident MI during the follow-up. Interaction terms between covariates and cognitive trajectories were added to explore potential risk factors for cognitive change after MI.

We further conducted propensity score (PS) matching to reduce the confounding bias. Participants who experienced a myocardial infarction (MI) during the follow-up (case) were randomly matched with (1:3) participants free of MI (control), using the nearest neighbor method without replacement and a caliper width of 0.2 of the standard deviation of the logit of the estimated PS (Chen et al., 2022). All cases matched successfully. We compared the baseline characteristics and standardized differences (Table 1, Supplementary Figures 2, 3) before and after matching (Yang and Dalton, 2012). Then, the primary analysis was repeated after matching.

**Table 1:** Baseline participant characteristics according to MI.

	**Incident MI (*n* = 421)**	**Control (*n* = 10,866)**	*p*-value before PS^*^	**Matched control (*n* = 1,263)**	***p*-value after PS**
**Continuous variables**					
Age	60.0 (9.0)	58.2 (9.2)	< 0.001	60.0 (9.4)	0.964
Number of IADLs	0.2 (0.7)	0.1 (0.6)	0.017	0.2 (0.6)	0.213
Episodic memory	3.2 (1.9)	3.3 (1.9)	0.746	3.3 (2.0)	0.908
Visuospatial abilities	0.6 (0.5)	0.7 (0.5)	0.813	0.6 (0.5)	0.741
Attention and calculation	2.9 (2.0)	2.8 (2.0)	0.178	2.8 (2.0)	0.198
Orientation	3.7 (1.4)	3.8 (1.4)	0.862	3.8 (1.4)	0.951
**Categorical variable**					
Males	172 (40.9)	5,252 (48.3)	0.003	546 (43.2)	0.393
**Education**			0.499		0.398
Illiterate	118 (28.0)	2,891 (26.6)		338 (26.8)	
Primary school	156 (37.1)	4,345 (40.0)		467 (37.0)	
Middle school	99 (23.5)	2,307 (21.2)		274 (21.7)	
High school and above	48 (11.4)	1,323 (12.1)		184 (14.6)	
**Marital status**			0.510		0.830
Married	368 (87.4)	9,713 (88.8)		1,190 (87.8)	
Other status	53 (12.6)	1,153 (11.3)		154 (12.2)	
**Residence**			0.019		0.791
Urban	102 (24.2)	2,128 (19.6)		298 (23.6)	
Rural	319 (75.6)	8,738 (80.4)		965 (96.4)	
Current smoking	147 (34.9)	4,254 (39.2)	0.081	457 (36.2)	0.639
Current drinking	88 (20.0)	3,161 (26.8)	0.002	263 (20.8)	0.702
Depression	111 (26.7)	2,075 (19.1)	< 0.001	332 (26.3)	0.987
Hypertension	173 (41.1)	2,778 (25.6)	< 0.001	511 (40.4)	0.819
Diabetes	51 (11.8)	709 (5.8)	< 0.001	174 (13.8)	0.385
High total cholesterol	89 (21.1)	1,460 (13.4)	< 0.001	263 (20.9)	0.890
Lung diseases	61 (14.5)	903 (8.3)	< 0.001	185 (14.7)	0.937
Cancer	5 (1.2)	88 (0.8)	0.394	16 (1.3)	0.899

^*^Calculated using *t-test* for continuous covariates and χ^2^ or Fisher test for categorical covariates.

CHARLS did not measure body mass index (BMI) in Wave 1. In sensitivity analysis, we further adjusted for BMI using BMI data in Wave 2 or Wave 3. Considering the strong association between stroke and cognitive decline, we excluded participants with stroke in the primary analysis and further included stroke patients in the sensitivity analysis (Xie et al., 2019; Hua et al., 2023). We restricted the analysis individuals who received cognitive assessments in all waves as a way to handle missing data.

Statistical analyses were performed from April 2023 to July 2023 using SAS version 9.4 TS1M7. A two-sided *p* < 0.05 was considered statistically significant.

## 3 Results

### 3.1 Baseline characteristics and cognitive tests

During a median of 7-year (interquartile range: 4–7 years) follow-up, 421 individuals [3.7% of 11,287 included individuals, mean (SD) age, 60.0 (9.0) years; 59.1% female] experienced incident MI. Baseline characteristics according to incident MI are shown in Table 1. The number of incident MI between each wave is shown in Supplementary Table 1. From waves 1–4, the available cognitive assessments were 421, 386, 383, and 300 in the MI group, and 10,866, 9,694, 9,420, and 7,511 in the control group (Supplementary Table 2). MI developed at a mean of 3.1 years (SD, 1.9 years) after baseline. The MI group received a mean number of 1.8 (SD, 0.8) cognitive tests before MI onset, and a mean number of 1.7 (SD, 0.9) cognitive tests after MI onset. Supplementary Figure 4 and Supplementary Table 3 shows the distribution of baseline cognitive scores. Characteristics of 1,148 (8.9%) participants who lost to follow-up are shown in Supplementary Table 4.

### 3.2 Difference between the MI group and the control group during the pre-MI period

For the control group without MI, scores of all cognitive domains declined during the whole follow-up period (Table 2 and Supplementary Figure 5). The baseline cognitive scores of the MI group were not different from the baseline scores of the control group. Meanwhile, the change rate (slope) of cognitive scores among the MI group during the pre-MI period was similar to that among the control group from baseline to the end of follow-up. In conclusion, the cognitive function of the MI group declined gradually before MI onset but was not weaker than the cognitive function of those without MI.

**Table 2:** Trends in cognitive function of the MI group and the control group before matching (ratio 1:3)^a,b^.

**Variables^c^**	**Global cognition**		**Episodic memory**		**Visuospatial ability**		**Orientation**		**Attention and calculation**	
	β **(95% CI)**	***p*** **-value**	β **(95% CI)**	***p*** **-value**	β **(95% CI)**	***p*** **-value**	β **(95% CI)**	***p*** **-value**	β **(95% CI)**	***p*** **-value**
Cognitive slope of control group	−0.036 (−0.038, −0.033)	< 0.001	−0.007 (−0.010, −0.003)	< 0.001	−0.037 (−0.041, −0.034)	< 0.001	−0.036 (−0.038, −0.033)	< 0.001	−0.052 (−0.055, −0.049)	< 0.001
Difference in baseline cognitive scores	0.074 (−0.007, 0.154)	0.073	0.027 (−0.069, 0.123)	0.582	0.016 (−0.079, 0.111)	0.742	0.032 (−0.048, 0.112)	0.428	0.070 (−0.021, 0.162)	0.129
Difference in cognitive slope before MI	−0.001 (−0.028, 0.025)	0.920	0.000 (−0.032, 0.032)	0.990	0.011 (−0.022, 0.043)	0.520	−0.008 (−0.035, 0.020)	0.581	0.015 (−0.014, 0.045)	0.306
Acute cognitive change after MI	0.013 (−0.085, 0.111)	0.792	0.023 (−0.096, 0.142)	0.703	−0.032 (−0.164, 0.100)	0.638	0.011 (−0.099, 0.120)	0.851	0.020 (−0.099, 0.139)	0.741
Change in cognitive slope after MI	−0.007 (−0.040, 0.027)	0.691	0.003 (−0.038, 0.043)	0.898	−0.021 (−0.065, 0.024)	0.355	0.008 (−0.026, 0.043)	0.635	−0.026 (−0.063, 0.011)	0.172

^a^Adjusted for baseline age, sex, education, marital status, residence, current smoking, current drinking, hypertension, dyslipidaemia, cancer, lung diseases, heart problems, depression, and number of IADLs.

^b^All cognitive values are z-score transformed.

^c^Detailed description for variables is shown in Supplementary Figure 1.

### 3.3 Difference between pre-MI and post-MI cognitive trends

Whether MI patients experienced acute cognitive change at MI onset was reflected by the estimate of the variable “Acute cognitive change,” while whether the cognitive slope in several years after MI was different from the slope before MI was reflected by the estimate of the variable “Change in cognitive slope after MI,” respectively (Table 2). Participants did not experience an acute cognitive change at the time of MI (global cognition score; β, 0.013 SD/year; 95% CI, −0.085 to 0.111; *p* = 0.792). The cognitive slope in the long time after MI was not statistically different from the pre-MI cognitive slope (global cognition score; β, −0.007 SD/year; 95% CI, −0.040 to 0.027; *p* = 0.691). Therefore, the cognitive decline rate post-MI was consistent with the pre-MI rate.

The conclusions after matching were constant with the conclusions before matching (Table 3 and Supplementary Table 5). MI was not associated with acute cognitive decline (global cognition score; β, 0.016 SD/year; 95% CI, −0.080 to 0.111; *p* = 0.747; Table 3) or accelerated long-term decline after MI (global cognition score; β, −0.006 SD/year; 95% CI, −0.039 to 0.026; *p* = 0.701).

**Table 3:** Trends in cognitive function of the MI group and the control group after matching (ratio 1:3)^a,b^.

**Variables^c^**	**Global cognition**		**Episodic memory**		**Visuospatial ability**		**Orientation**		**Attention and calculation**	
	β **(95% CI)**	***p*** **-value**	β **(95% CI)**	***p*** **-value**	β **(95% CI)**	***p*** **-value**	β **(95% CI)**	***p*** **-value**	β **(95% CI)**	***p*** **-value**
Cognitive slope of control group	−0.035 (−0.042, −0.028)	< 0.001	−0.004 (−0.014, 0.005)	0.360	−0.041 (−0.051, −0.031)	< 0.001	−0.036 (−0.043, −0.028)	< 0.001	−0.051 (−0.059, −0.043)	< 0.001
Difference in baseline cognitive scores	0.087 (0.002, 0.173)	0.045	0.036 (−0.068, 0.140)	0.495	0.016 (−0.088, 0.120)	0.762	0.031 (−0.055, 0.117)	0.483	0.089 (−0.010, 0.187)	0.078
Difference in cognitive slope before MI	−0.003 (−0.029, 0.023)	0.814	−0.003 (−0.036, 0.029)	0.846	0.016 (−0.018, 0.050)	0.357	−0.008 (−0.036, 0.020)	0.558	0.013 (−0.017, 0.043)	0.387
Acute cognitive change after MI	0.016 (−0.080, 0.111)	0.747	0.026 (−0.092, 0.143)	0.670	−0.036 (−0.168, 0.095)	0.589	0.013 (−0.095, 0.121)	0.811	0.025 (−0.092, 0.142)	0.672
Change in cognitive slope after MI	−0.006 (−0.039, 0.026)	0.701	0.003 (−0.037, 0.043)	0.870	−0.022 (−0.066, 0.022)	0.332	0.009 (−0.025, 0.043)	0.620	−0.025 (−0.062, 0.012)	0.183

^a^Adjusted for baseline age, sex, education, marital status, residence, current smoking, current drinking, hypertension, dyslipidaemia, cancer, lung diseases, heart problems, depression, and number of IADLs.

^b^All cognitive values are z-score transformed.

^c^Detailed description for variables is shown in Supplementary Figure 1.

**Figure 2:**
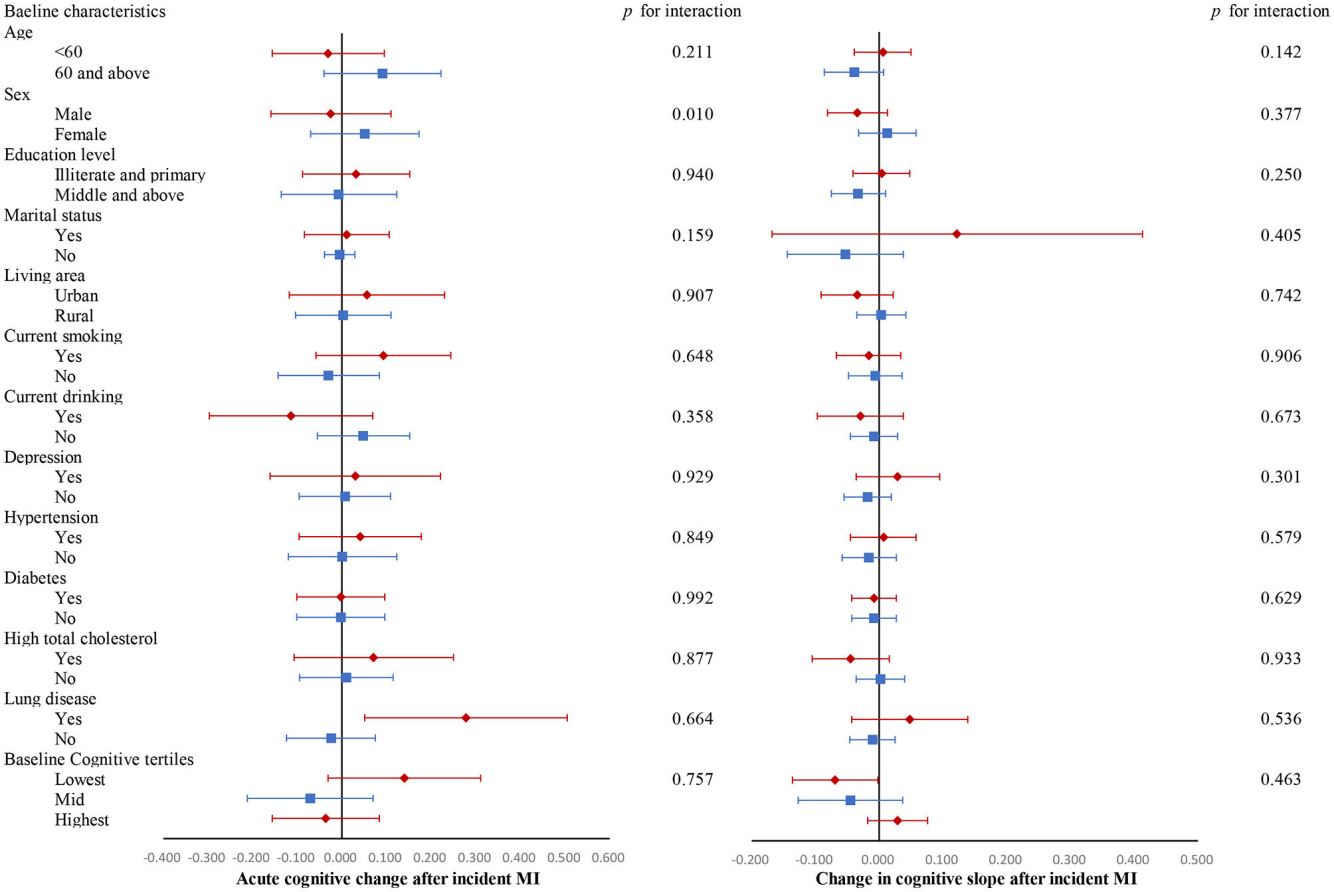
Changes in global cognitive trajectories after MI compared to the pre-MI period according to subgroups. Adjusted for baseline age, sex, educational level, marital status, area of residence, smoking, drinking, number of instrumental activities of daily living (IADLs), depression, hypertension, diabetes, high total cholesterol, lung diseases, and cancer. All cognitive values are *z*-score transformed.

In most of the subgroups, incident MI was not associated with acute cognitive change at the time of MI or changes in cognitive slope in the years following MI (Figure 2). Supplementary Figure 6 shows changes in each domain-specific cognitive trajectory after MI compared to the pre-MI period according to subgroups. The cognitive function of MI participants with lung disease acutely increased at the time of MI in domains of global cognition and visuospatial abilities. Participants in the median and highest baseline cognitive tertiles did not exhibit changes in post-MI cognitive decline, while those in the lowest baseline cognitive tertile showed an accelerated decline in global cognition score after MI (β, −0.069 SD/year; 95% CI, −0.136 to −0.002; *p* = 0.047).

### 3.4 Sensitivity analysis

To assess the stability of our results, after restricting the study to participants who had cognitive data in all four waves, the MI group had higher baseline scores in the attention and calculation domain but still had non-significant acute cognitive change or post-MI cognitive slope change, which is in accordance with our primary results. In other sensitivity analyses, the MI group showed similar pre-MI cognitive slope compared with the control group. MI group also did not show acute cognitive change or changes in cognitive slope after MI (Supplementary Tables 6–8).

## 4 Discussion

Among 11,287 participants in this community-based cohort study, the MI group did not show impaired cognitive function before the onset of MI. MI was not associated with acute cognitive decrement at the time of MI or accelerated cognitive deterioration in several years following MI.

Previous studies learning the risk of cognitive impairment of CHD patients yield mixed results (Schievink et al., 2017). A meta-analysis identified 28 articles and showed a pooling result that CHD increased the risk of all-cause cognitive impairment (RR = 1.27), while 13 of them reported insignificant association (Liang et al., 2021). Due to the hospital-based design, previous works rarely compared cognitive function after CHD with the function before CHD. Evidence from the Chinese population is scarce (Xie et al., 2019; Johansen et al., 2023). To the best of our knowledge, two articles have learned the trends in cognition before and after CHD. Xie et al. (2019) analyzed participants in the UK from the English Longitudinal Study of Aging (ELSA) and unveiled that CHD was not associated with pre-CHD cognitive disadvantage or acute cognitive change. By pooling six cohort studies from the US, Johansen et al. (2023) also revealed that MI patients had similar cognitive trajectories before MI compared with the without-MI participants and did not suffer from acute cognitive decline. These results are constant with our findings. However, our results in cognitive slopes in years after MI were different from the above two articles. Xie et al. (2019) disclosed that the decline rate accelerated after incident CHD in domains of global cognition, memory, and orientation, but not semantic fluency. Johansen et al. (2023) narrated that the first MI was correlated with faster declines in memory, executive function, and global cognition over time and that the results differed by race and sex. Notably, Whitlock et al. (2021) reported that the cognitive slope before and after coronary artery bypass grafting (CABG) and percutaneous coronary intervention (PCI) were similar in the Health and Retirement Study (HRS) in US.

At least five studies had learned the timeline of cognitive decline before and after stroke (Levine et al., 2015; Zheng et al., 2019; Eng et al., 2021; Heshmatollah et al., 2021; Hua et al., 2023). A previous CHARLS study discovered that stroke was associated with acute cognitive decline in the short term after stroke and accelerated cognitive decline in the future among the Chinese population (Hua et al., 2023). Studies learning the European population further indicated that stroke participants had shown faster cognitive decline even before stroke, suggesting cognitive decline as a predictor for stroke onset (Zheng et al., 2019; Heshmatollah et al., 2021). Previous studies connected the pre-disease cognitive decline to the effect of vascular risk factors which is hard to be adjusted in the statistical models (Whalley et al., 2006; Zheng et al., 2019). Considering that MI and stroke are both vascular diseases, it is tempting to postulate that MI patients had cognitive disadvantages before MI and experienced steeper annual cognitive decline after MI.

Why the decline rate of cognitive function of the stroke population was steeper than that of the without-stroke population during the pre-stroke period, but the decline rate of the MI group was similar to the rate of the without-MI group during the pre-MI period? Although stroke and MI mainly result from atherosclerosis and atrial fibrillation, the pre-disease cognitive patterns are different. Let us make a bold assumption. The pre-stroke cognitive disadvantage is not because of vascular risk factors (e.g., diabetes, smoking, or atherosclerosis) but because of the intracranial damage (e.g., subclinical brain infarcts and white matter hyperintensity) (Dhamoon et al., 2018). We tried to eliminate the baseline imbalance, including cardiovascular risk factors, by PS matching. However, these confounders are sometimes time-varying and thus hard to adjust if we only consider the baseline time-point (Hernan et al., 2000; Robins et al., 2000). If our hypothesis were correct, performing head magnetic resonance imaging (MRI) might predict pre-stroke cognitive decline and onset of stroke.

Our study added to prior stroke research which learned the timeline of cognitive decline after stroke. It is widely reported that the incident rate of dementia within 6 months after a stroke was much higher than the incident rate several years after stroke (Pendlebury et al., 2019). The concept, named “acute cognitive change/decline” in the linear mixed model, could explain this phenomenon, and it is obviously caused by the strike of acute illness inside the brain (Baron et al., 1981; Garcia-Alloza et al., 2011). The absence of acute cognitive change after MI supported that the effect of MI on the brain happened gradually rather than suddenly.

This is one of the largest important cohort studies reporting the cognitive trends before and after MI. Another advantage of our study is the study population. Chinese participants have different economic and educational levels, which would lead to different patterns of cognitive decline after the illness (Levine et al., 2018). However, several limitations need to be acknowledged. Firstly, CHARLS did not differentiate the type of heart problems in the first wave and only recorded MI in the latter waves. This prevented us from considering subgroups of atrial fibrillation, angina, or asymptomatic CHD. We were also unable to investigate the prognosis of different MI treatments (conservative internal medical treatment, PCI, and CABG). Fortunately, recording all kinds of heart problems enabled a strict exclusion criterion during sample selection. Second, the sensitivity and specificity of self-reported MI in CHARLS are unknown. Part of the participants would mistake unstable angina or arrhythmias for MI (Rosamond et al., 1995). Lower positive predictive values for self-reported MI would underestimate the negative effect of MI on cognition. Taking baseline cognitive function into account helps improve the sensitivity and specificity of self-reported diagnosis of MI. However, higher baseline cognitive scores represent better cognitive reserve, which provides a buffer against the damage of MI on the brain. Those with higher baseline scores would show slower cognitive decline, although they have higher MI diagnostic accuracy. Third, not all cognitive tasks are created equal. Each domain-specific cognitive test in CHARLS is relatively simple. We generated a global cognition by summing the *z*-scores of four tests. The aggregated measure makes the results more readable, however, it could not reflect the overall brain function. Mini-mental state examination (MMSE) or Montreal Cognitive Assessment (MoCA) can diagnose MCI and dementia by using cut-off points of the sum score, while the global cognition score in our research lacks this capability (Folstein et al., 1975; Jia et al., 2021).

In conclusion, the cognitive function of the MI group was not poorer than the without-MI group before MI onset. MI was not associated with acute cognitive decline in the short term after MI or accelerated cognitive decline in the years following MI. The cognitive pattern before an incident MI conflicted with that before an incident stroke. Shared cardiovascular risk factors for MI and stroke could not explain the pre-stroke cognitive discrepancy. The mechanism underlying the difference is unknown and warrants future study.

## Data availability statement

The raw data supporting the conclusions of this article will be made available by the authors, without undue reservation.

## Ethics statement

The studies involving humans were approved by Institutional Review Board at Peking University. The studies were conducted in accordance with the local legislation and institutional requirements. The participants provided their written informed consent to participate in this study. Written informed consent was obtained from the individual(s) for the publication of any potentially identifiable images or data included in this article.

## Author contributions

SZ: Writing – original draft, Conceptualization. JS: Writing – original draft, Funding acquisition, Writing – review & editing. JD: Writing – original draft, Data curation, Formal analysis, Methodology, Software, Validation, Visualization. QC: Project administration, Resources, Writing – review & editing, Funding acquisition, Supervision, Validation. JH: Conceptualization, Data curation, Formal analysis, Methodology, Project administration, Resources, Software, Visualization, Writing – original draft, Writing – review & editing.
